# Left Ventricular Global Longitudinal Strain (GLS) Is a Superior Predictor of All-Cause and Cardiovascular Mortality When Compared to Ejection Fraction in Advanced Chronic Kidney Disease

**DOI:** 10.1371/journal.pone.0127044

**Published:** 2015-05-15

**Authors:** Rathika Krishnasamy, Nicole M. Isbel, Carmel M. Hawley, Elaine M. Pascoe, Matthew Burrage, Rodel Leano, Brian A. Haluska, Thomas H. Marwick, Tony Stanton

**Affiliations:** 1 Department of Renal Medicine, The University of Queensland at Princess Alexandra Hospital, Brisbane, Australia; 2 School of Medicine, The University of Queensland, Brisbane Australia; 3 Cardiovascular Imaging Research Centre, The University of Queensland at Princess Alexandra Hospital, Brisbane, Australia; 4 Menzies Research Institute, University of Tasmania, Hobart, Australia; University of Perugia, ITALY

## Abstract

**Background:**

Echocardiographic global longitudinal strain (GLS) is increasingly recognised as a more effective technique than conventional ejection fraction (EF) in detecting subtle changes in left ventricular (LV) function. This study investigated the prognostic value of GLS over EF in patients with advanced Chronic Kidney Disease (CKD).

**Methods:**

The study included 183 patients (57% male, 63% on dialysis) with CKD stage 4, 5 and 5Dialysis (D). 112 (61%) of patients died in a follow up of 7.8 ± 4.4 years and 41% of deaths were due to cardiovascular (CV) disease. GLS was calculated using 2-dimensional speckle tracking and EF was measured using Simpson’s biplane method. Cox proportional hazard models were used to assess the association of measures of LV function and all- cause and CV mortality.

**Results:**

The mean GLS at baseline was -13.6 ± 4.3% and EF was 45 ± 11%. GLS was a significant predictor of all-cause [Hazard Ratio (HR) 1.09 95%; Confidence Interval (CI) 1.02–1.16; p = 0.01] and CV mortality (HR 1.16 95%; CI 1.04–1.30; p = 0.008) following adjustment for relevant clinical variables including LV mass index (LVMI) and EF. GLS also had greater predictive power for both all- cause and CV mortality compared to EF. Impaired GLS (>-16%) was associated with a 5.6-fold increased unadjusted risk of CV mortality in patients with preserved EF.

**Conclusions:**

In this cohort of patients with advanced CKD, GLS is a more sensitive predictor of overall and CV mortality compared to EF. Studies of larger populations in CKD are required to confirm that GLS provides additive prognostic value in patients with preserved EF.

## Introduction

Left ventricular (LV) systolic dysfunction, most commonly assessed by echocardiographic ejection fraction (EF) is an established predictor of outcome. It is most widely used as a prognostic marker in patients with heart failure and determines eligibility for cardiac interventions. Observational studies in chronic kidney disease (CKD) have demonstrated an association between impaired EF with a greater risk of cardiovascular (CV) and all-cause mortality[1–3]. Despite a high prevalence of CV insults and progressive symptoms of heart failure, EF remains preserved in the majority of patients with CKD [4–6]. In addition, several studies have identified less than a third of patients with end-stage kidney disease (ESKD) to have detectable systolic dysfunction[1, 7, 8]. Whilst this discrepancy is related to the complex pathophysiology of CV disease in CKD, technical limitations of EF measurement may also be a contributing factor. Standard echocardiographic EF measurement requires accurate tracing of endocardial border and is operator, volume and load dependent [9] resulting in a limited reproducibility [10]. There is a growing interest in the current literature for other non-invasive and more objective assessment of LV function. This is of particular relevance to the CKD cohort who undergo progressive cardiac remodelling.

Global Longitudinal Strain (GLS) assessed using automated speckle-tracking echocardiography (STE) is an emerging technique for detecting and quantifying subtle disturbances in LV systolic function. GLS reflects the longitudinal contraction of the myocardium and its accuracy has been validated against tagged magnetic resonance imaging (MRI)[11]. This method is operator independent, more reproducible than EF, easily measured and integrated to standard echocardiogram method[12]. In the general population and patients with heart failure, GLS was shown to be a superior predictor of cardiac events and all-cause mortality compared to EF [13, 14]. More recently, GLS was found to be a robust prognostic marker following myocardial infarction[15] and cardiac surgery[16], and in patients with cardiomyopathy[17] and aortic stenosis[18].

There is limited data relating to the utility and prognostic importance of strain in comparison to EF in CKD. We reported GLS provided an incremental prognostic power over relevant clinical and echocardiographic measurements in predicting overall survival in patients with mild to moderate CKD[19]. More recently, Kramann et al demonstrated strain parameters were independent risk factors for CV and all-cause mortality in 171 dialysis patients with 2.5 years of follow-up[20]. This current long-term study aimed to assess the prognostic value of GLS over conventional EF on both CV and all-cause mortality in patients with stage 4, 5 and 5D CKD. The study hypothesized that reduced GLS at baseline echocardiography may be associated with increased risk of all-cause and cardiovascular mortality in patients with advanced CKD.

## Materials and Methods

This was a cardiac imaging sub-study of a larger randomized controlled trial, Longitudinal Assessment of Numerous Discrete Modifications of Atherosclerotic Risk factors in Kidney disease (LANDMARK) 1 study powered for vascular structure and function end-points. LANDMARK 1 evaluated the efficacy of a nurse-driven multiple risk factor intervention over conventional care in reducing atheroma burden and endothelial dysfunction in patients with stage 4,5 and 5D CKD[21]. From 1999 to 2001, 200 patients were recruited in a single centre study involving detailed assessment of cardiac imaging in patients with CKD. The inclusion criteria were patients > 18 years of age, stage 4 and 5 CKD with a calculated glomerular filtration rate (GFR) of ≤ 30mL/min using the Cockcroft–Gault equation[22] or on maintenance dialysis therapy (hemodialysis or peritoneal dialysis for at least 3 months). Patients with pre-existing conditions which were expected to limit their life expectancy to < 6 months were excluded. The study protocol was approved by Human Ethics Committee of the University of Queensland and Princess Alexandra Hospital. All participants gave written, informed consent to participate in the study. Following 2 years of intervention, the LANDMARK 1 study did not achieve the primary outcome of regression of carotid intima media thickness (c-IMT) or improvement in brachial artery reactivity (BAR)[21]. 183 patients with follow-up data on CV and all-cause mortality were included in this cardiac imaging sub-study.

### Clinical Assessment

Demographic data, including an assessment of risk factor status and history of cardiovascular disease were recorded; cardiovascular medications were documented and a 12-lead electrocardiogram was reviewed. Blood pressure, averaged from 3 seated measurements taken after a 5-minute rest, was measured pre dialysis on a short (2-day) break in all hemodialysis participants. Hypertension and hypercholesterolemia were defined by the use of antihypertensive or lipid-lowering therapy, respectively. Diabetes was defined by a history of this diagnosis or use of oral hypoglycemic agents or insulin. Previous cardiovascular event was defined as a history of documented myocardial infarction, coronary artery bypass surgery, percutaneous coronary intervention, or hospital admission with acute coronary syndrome (ischemic chest pain and/or electrocardiographic [ECG] changes suggestive of ischemia with no elevation in cardiac enzymes), peripheral vascular disease including peripheral revascularization procedure or amputation due to ischemia.

### Biochemical assessment

Blood for biochemical analyses was obtained from fasting venous samples and taken at baseline. Serum total cholesterol, triglycerides, albumin, phosphate, parathyroid hormone (PTH), corrected calcium, creatinine, and hemoglobin concentration were measured using standard laboratory techniques.

### Echocardiography

All echocardiographic parameters were measured offline in batches by two experienced observers blinded to clinical and outcome data. Timing of echocardiogram for hemodialysis participants were standardised and performed immediately prior to dialysis on a short (2-day) break. Intra- and inter-observer variation were assessed by intra-class correlation co-efficient (ICC) and compared using Z-scores and Bland Altman plots and have been published elsewhere [19].

#### a) Two-Dimensional (2D) Echocardiography

Cine loops from 3 standard apical views (4-chamber, 2-chamber, and apical long-axis) were recorded using gray-scale harmonic imaging and saved in raw data format (Vivid 7, General Electric Medical Systems, Horten, Norway). Images were obtained at a frame rate of 50 to 70 per second, and digital loops were saved onto optical disc for off-line analysis (EchoPac 8.0, General Electric Medical Systems). End-diastolic and end-systolic volumes were used to calculate EF by Simpson biplane method from the apical 4- and 2-chamber views[23]. Preserved left ventricular EF was defined as ≥50%[24, 25]. LV mass was calculated with the formula: LV mass = 0.8 × {1.04[([LV internal dimension + septal wall thickness + posterior wall thickness]^3^ − LV internal dimension^3^)] + 0.6 g. Left ventricular mass was indexed to height^2.7^ and left ventricular hypertrophy (LVH) was defined as ≥51 g/m^2.7^ for both sexes[26]. LVH measurement was not available for 20 patients. The effect of afterload and preload on GLS was evaluated using LV wall stress and LV end diastolic volume (LVEDV). LV meridional wall stress was assessed using validated formula: LV wall stress = [0.334 x systolic BP x LV end diastolic diameter]/ [LV wall thickness in end diastole x (1 +LV wall thickness in end diastole /LV end diastolic diameter)] dynes/cm^2^ x 1000[27].

#### b) 2D Speckle- Strain

The endocardial borders were traced in the end-systolic frame of the 2D images from the 3 apical views. Speckles were tracked frame by-frame throughout the LV wall during the cardiac cycle and basal, mid, and apical regions of interest were created. Segments that failed to track were manually adjusted by the operator. GLS was calculated as the mean strain of 18 segments. Index beat technique was used to measure GLS in 10 patients with atrial fibrillation (AF). This method has been validated and well described by Kusunose et al[28] and Su et al[29] as a reliable way to assess GLS in patients with AF. Previous studies have demonstrated that healthy individuals have GLS ranging from -16 to -19% [30, 31]. A cut off at -16% has been shown to provide important risk stratification and prognostic value[16]. Therefore, in our study we defined impaired GLS as>-16%(a less negative value reflects a more impaired GLS).

### Follow-up

Follow-up was obtained by clinic review, or for patients living remotely, by telephone contact and Australia and New Zealand Dialysis and Transplant (ANZDATA) Registry review. Record was made of those patients who had undergone renal transplantation and those who remained on renal dialysis. All-cause mortality was defined as death from any cause. Average length of follow up was 7.8± 4.4 years. Deceased patients were identified from the medical records at Princess Alexandra Hospital, Brisbane, Australia and ANZDATA registry and were matched according to first name, last name, identification number and date of birth. Individuals with incomplete data were contacted through general practitioners or telephone interview with their families and were excluded if data was missing (17 patients). One hundred and twelve deceased patients were identified. All other patients were considered to be alive at the end of the follow-up period. Cause of cardiac death was reported by the patient’s attending nephrologist according to the following categories: myocardial ischemia (presumed), myocardial ischemia/infarction, cardiac failure, cerebrovascular accident and sudden cardiac death [including arrhythmia and cardiac arrest (cause uncertain, whether in or out of hospital)]. Forty six cardiac deaths were identified.

### Statistical analysis

Baseline characteristics at the entry of the study were expressed as frequencies and percentages for categorical variables, mean±standard deviation (SD) for continuous, normally distributed variables and median [interquartile range] for continuous, non-normally distributed variables. The relationship between GLS and variables of interest were assessed using Pearson correlation coefficients for continuous normally distributed variables and Spearman’s correlation for categorical or non–normally distributed data.

Unadjusted CV and all-cause mortality rates and incidence rate ratios (IRR) for EF and GLS were calculated using Poisson regression and survival estimates were determined using the Kaplan-Meier method. Univariate analysis was performed to assess relationships between baseline demographic, biochemical, echocardiographic parameters and the clinical outcomes of CV and all-cause mortality. Cox proportionate hazard models were used to determine significant predictors of all-cause and CV mortality. Multivariate regression analysis included all univariate variables with p<0.1 and with adjustment for systolic BP as a lockterm. Systolic BP has been shown to be a relevant source of variation to GLS readings[32]. Treatment allocation for the LANDMARK 1 study was included in this analysis.

To further compare the predictive value of EF and GLS, nested cox models with separate addition of EF and GLS to a baseline model containing significant demographic and biochemical co-variates were constructed. The independence and incremental value of each measure of LV function over baseline was assessed using likelihood-ratio tests. Model discrimination was further assessed using Harrell’s C-statistic[33]. Interaction terms were examined for GLS and other significant predictors of mortality. Proportional hazards assumptions were assessed graphically and by formal tests including Schoenfeld’s test. Data were analyzed using Stata (version 12; StataCorp LP, College Station, Tx). P values less than 0.05 were considered statistically significant for all described analyses.

### Sensitivity analyses

Competing-risks regression was performed to adjust for renal transplantation during follow-up as a competing risk in the survival analyses. As the definition of preserved EF has been variably classified as either > 45% or > 50%[34], mortality rates and survival estimates were also calculated using an EF cut-off of 45%.

## Results

### Clinical and echocardiographic characteristics

183 patients were followed up for an average length of 7.8 ± 4.4 years. The clinical and echocardiographic characteristics of patients are presented in Table 1. Participants were predominantly male with a mean age of 55±15 years and had a high prevalence of hypertension, hypercholesterolemia and history of smoking. At baseline, 40% were receiving hemodialysis and 23% were receiving peritoneal dialysis and 37% of patients had not yet commenced renal replacement therapy. During the follow-up period, 38% of the total study population received a renal transplant and 75% of patients with stage 4/5 CKD at baseline commenced dialysis. The median duration on dialysis during follow-up was 1.8 years (Interquartile Range of 0.5–4.0). In this cohort, mean EF was 45 ± 11% and mean GLS was -13.6 ± 4.3%. There were no differences in GLS value between patients with stage 4/5 CKD (mean GLS-13.9 ± 4.3) compared to those established on dialysis (mean GLS -13.4 ± 4.3, p = 0.4). Left ventricular hypertrophy (LVH) was identified in 53% of the study population. GLS was significantly correlated with EF (r = -0.6, p<0.001) and LVMI (r = 0.3, p<0.001) at baseline.

**Table 1:** Baseline characteristics of 183 participants.

**Variable**	**Mean (±SD) or number (%)**
Age (years)	55± 15
Male	105(57%)
Diabetes mellitus	52(28%)
Hypertension	165(90%)
Smoking (current or former)	92(51%)
Hypercholesterolemia	98(54%)
Previous cardiovascular events	82(45%)
Body mass index (BMI) (kg/m^2^)	27±5
**Blood Pressure (BP)**	
Systolic BP (mmHg)	145±22
Diastolic BP (mmHg)	82±13
**Causes of renal disease**	
Diabetic nephropathy	41(22%)
Chronic GN	44(24%)
Renovascular /hypertensive nephrosclerosis	15(8%)
Reflux nephropathy	12(7%)
Adult Polycystic Kidney Disease (ADPKD)	18(10%)
Others	34(19%)
Unknown	19(10%)
**Renal factors**	
Stage 4/5 CKD (GFR<30ml/min)	68(37%)
Hemodialysis	73(40%)
Peritoneal Dialysis	42(23%)
Duration of dialysis (y)(median and IQR)	1.8(0.5–4.0)
Received Renal Transplant	70(38%)
**Biochemistry**	
Corrected Calcium (mmol/L)	2.4±0.2
Phosphate (mmol/L)	1.8±0.5
Parathyroid Hormone (pmol/L) (median and IQR)	30(14.5–59)
Albumin(g/L)	38±5.2
Hemoglobin(g/L)	109±15
**Medication**	
ACE inhibitor	73(40%)
Β —Blocker	60(33%)
Calcium channel blocker	85(46%)
Cholesterol lowering agent	102(56%)
**Echocardiographic characteristics**	
LV end diastolic volume (mm)	140± 48
LV end systolic volume (mm)	79±37
LV mass index (g/m^2.7^)	55±17
LV hypertrophy (LVMI > 51 g/m^2.7^)	87(53%)
LV meridional systolic wall stress (dynes/cm^2^x1000)	149.8 ± 48.5
Ejection Fraction (%)	45±11
Global Longitudinal Strain (%)	-13.6±4.3

SD = standard deviation; CKD = chronic kidney disease; LV = left ventricular; LVMI = left ventricular mass index; GN = glomerulonephritis; ACE = angiotensin converting enzyme

### Association of GLS and EF with all-cause and CV mortality

**Fig 1:**
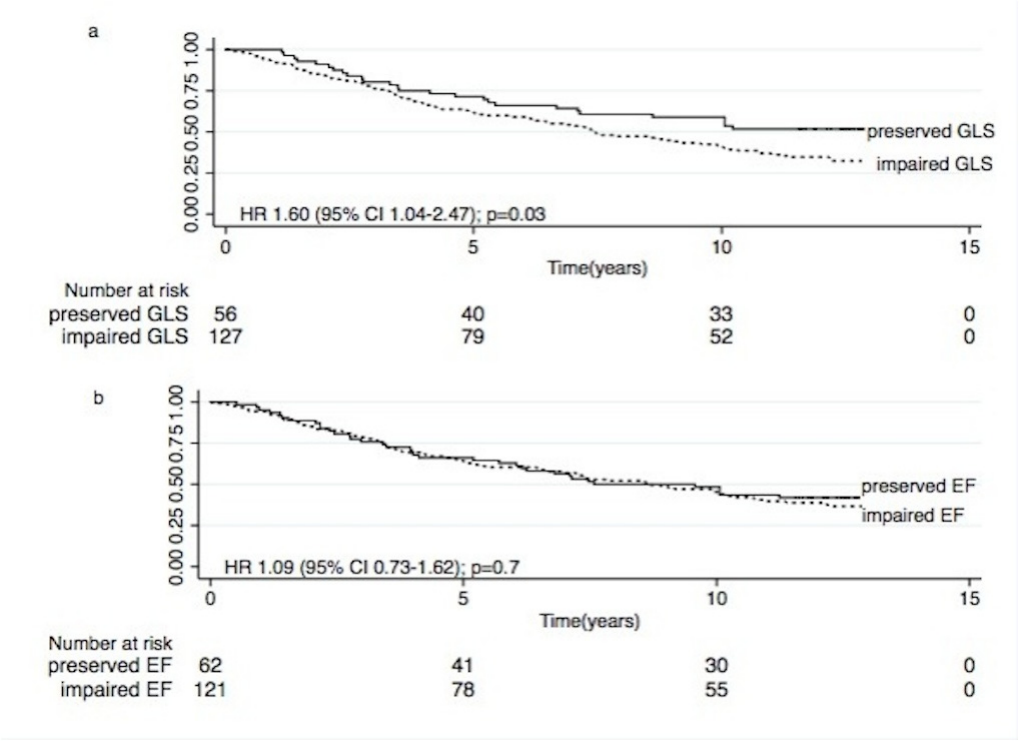
(a +b). Kaplan Meier all-cause survival estimates according to GLS (a) and EF (b).

**Fig 2:**
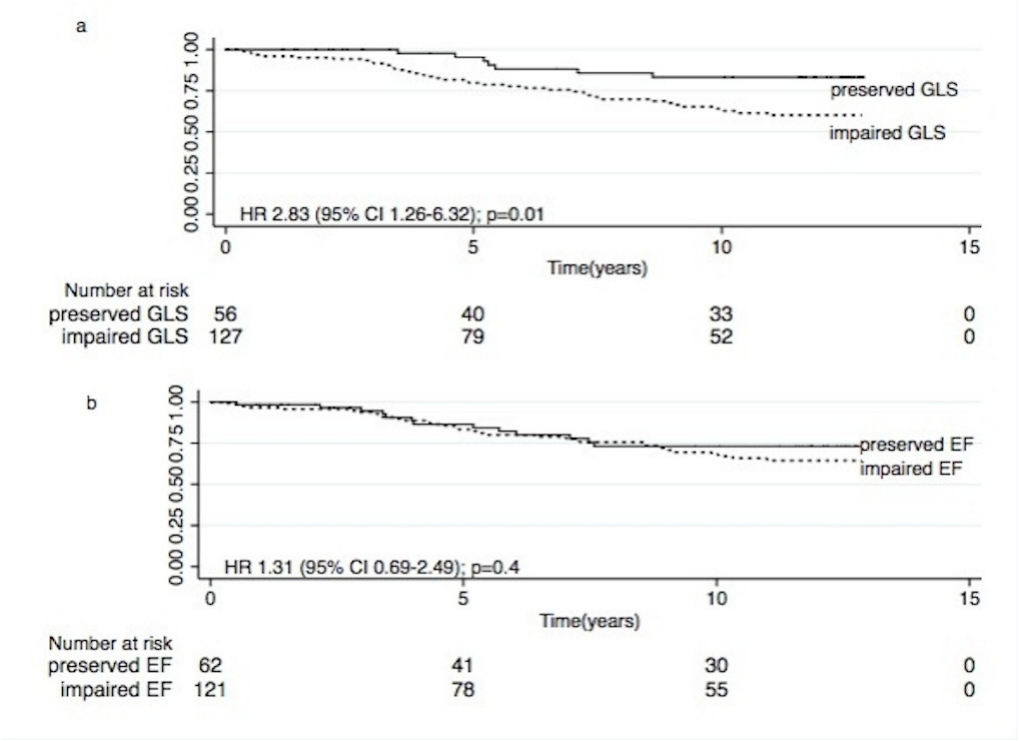
(a +b). Kaplan Meier CV survival estimates according to GLS (a) and EF (b).

Over 7.8 years of follow up, there were 46 (41.0%) cardiovascular deaths among the 112 (61.2%) fatal events. This includes cardiac arrest (50.0%), presumed myocardial ischemia (17.4%), myocardial ischemia and infarction (23.9%), cardiac failure (4.4%) and cerebrovascular accident (4.3%). The all-cause and CV mortality rates were substantially higher for patients with impaired GLS (GLS > -16%) compared to preserved GLS (GLS≤ -16%) (All–cause: 9.0 versus 5.6 per 100 person-years p = 0.01; CV: 4.1 versus 1.4 per 100 person-years p = 0.003) (Table 2). There was no difference in mortality rates among patients with preserved or impaired EF. Kaplan-Meier survival estimates for overall and CV survival dichotomized according to GLS (≤-16% and >-16%) and EF (≥50% and <50%) respectively are reported in Figs 1 and 2. Importantly, impaired GLS (>-16%) was associated with increased all-cause [log rank χ^2^ = 4.6, Hazard Ratio (HR) 1.60 95% Confidence Interval (CI) 1.04–2.47), p = 0.03] and CV mortality (log rank χ^2^ = 7.0, HR 2.83 95% CI 1.26–6.32 p = 0.01) compared with preserved GLS. In contrast, EF was not predictive of all-cause or CV deaths.

**Table 2:** CV and all-cause mortality rates according to LV function.

Event	Rate per 100 Person-Years (95%CI)					
	Preserved GLS	Impaired GLS	IRR, p value	PreservedEF	Impaired EF	IRR, p Value
All-cause mortality	5.6(3.8–8.1)	9.0(7.3–11.2)	1.6(1.0–2.6),0.01	7.4(5.3–10.3)	8.1(6.5–10.1)	1.0(0.7–1.7),0.3
CV mortality	1.4(0.7–3.0)	4.1(3.0–5.7)	2.9(1.3–7.6),0.003	2.7(1.5–4.6)	3.5(2.5–4.9)	0.2(0.7–2.7),0.2

CV = cardiovascular; GLS = global longitudinal strain; EF = ejection fraction; IRR = incidence rate ratio

### Predictors of all-cause and CV mortality

The univariate predictors of all–cause mortality were age, diabetes mellitus, smoking history, previous cardiovascular events, systolic and diastolic blood pressure (BP), GLS and LV mass index (LVMI) (Table 3). CV deaths were associated with age, male gender, diabetes mellitus, previous cardiovascular events, systolic BP, LV wall stress, LVEDV, LVMI and GLS (Table 4). Following adjustment with multivariable analysis, worsening GLS remained a significant predictor of all-cause mortality (HR 1.09 95% CI 1.02–1.16 p = 0.01) and CV mortality (HR 1.16 95% CI 1.04–1.30, p = 0.008). In these analyses, preload and afterload indices of LVEDV and LV wall stress were included to further elucidate loading effects on GLS.

**Table 3:** Cox univariate and multivariate regression analyses for predictors of all-cause mortality.

Variables	Univariate Analysis		Multivariate Analysis	
	HR (95% CI)	p value	HR (95% CI)	p value
Age (years)	1.05(1.04–1.07)	<0.001	1.07(1.05–1.09)	<0.001
Male gender	1.10(0.76–1.61)	0.5		
Diabetes Mellitus	1.66(1.12–2.46)	0.01		
Smoking history	1.50(1.02–2.20)	0.04	1.51(0.93–2.30)	0.1
Previous CV Events	2.77(1.89–4.06)	<0.001		
Systolic BP(mmHg)	1.01(1.00–1.02)	0.04	1.00(0.99–1.01)	0.6
Diastolic BP(mmHg)	0.98(0.97–1.00)	0.02		
BMI (kg/m^2^)	0.98(0.94–1.02)	0.4		
Duration on Dialysis	1.01(0.95–1.07)	0.7		
Treatment allocation	0.90(0.62–1.30)	0.6		
PTH (pmol/L)	1.01(0.99–1.01)	0.1	1.01(1.00–1.01)	0.005
Phosphate	1.03(0.71–1.49)	0.9		
GLS (%)	1.10(1.05–1.15)	<0.001	1.09(1.02–1.16)	0.01
EF (%)	0.98(0.63–1.00)	0.05	1.02(0.99–1.05)	0.09
LVMI (g/m^2.7^)	1.02(1.01–1.03)	0.001	1.02(1.00–1.04)	0.03
LVEDV (mm)	1.00(1.00–1.01)	0.6		
LV wall stress (dynes/cm^2^x1000)	1.00(1.00–1.01)	0.7		

CV = cardiovascular; BP = blood pressure; BMI = body mass index; PTH = parathyroid hormone; GLS = global longitudinal strain; EF = ejection fraction; LVMI = left ventricular mass index; LVEDV = left ventricular end diastolic volume; LV = left ventricular; HR = hazard ratio; CI = confidence interval

**Table 4:** Cox univariate and multivariate regression analyses for predictors of cardiovascular mortality.

Variable	Univariate Analysis		Multivariate Analysis	
	HR (95%CI)	p value	HR (95%CI)	p value
Age (years)	1.04(1.02–1.07)	<0.001	1.05(1.02–1.08)	<0.001
Male gender	1.97(1.04–3.75)	0.04	2.16(1.08–5.16)	0.03
Diabetes mellitus	3.03(1.70–5.43)	<0.001	2.55(1.25–5.20)	0.01
Smoking history	1.76(0.96–3.24)	0.06		
Previous CV Events	4.2(2.23–7.75)	<0.001		
Systolic BP (mmHg)	1.02(1.00–1.03)	0.03	1.01(0.99–1.03)	0.3
Diastolic BP (mmHg)	0.98(0.97–1.00)	0.2		
BMI (kg/m^2^)	1.03(0.98–1.09)	0.3		
Duration on Dialysis	1.01(0.92–1.10)	0.8		
Treatment allocation	1.15(0.64–2.05)	0.6		
GLS (%)	1.14(1.07–1.22)	<0.001	1.16(1.04–1.30)	0.008
EF (%)	0.97(0.95–1.00)	0.08	1.04(0.99–1.05)	0.08
LVMI (g/m^2.7^)	1.03(1.01–1.04)	0.003	1.03(0.99–1.05)	0.08
LVEDV (mm)	1.01(1.00–1.02)	<0.001		
LV wall stress (dynes/cm^2^ x1000)	1.01(1.00–1.01)	0.02		

Abbreviations as explained above

**Fig 3:**
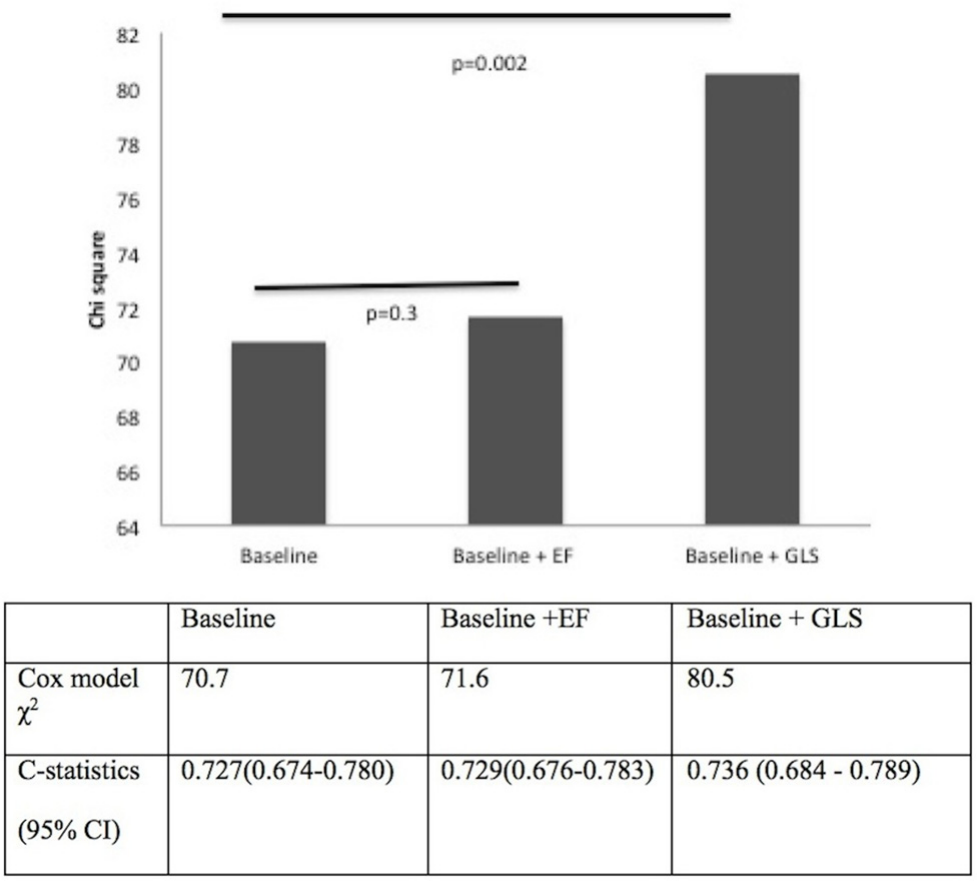
All-cause mortality: Comparing incremental value of EF and GLS to relevant demographic and biochemical variables (baseline model: age, diabetes mellitus, previous cardiovascular event, smoking history, systolic BP, diastolic BP, PTH).

**Fig 4:**
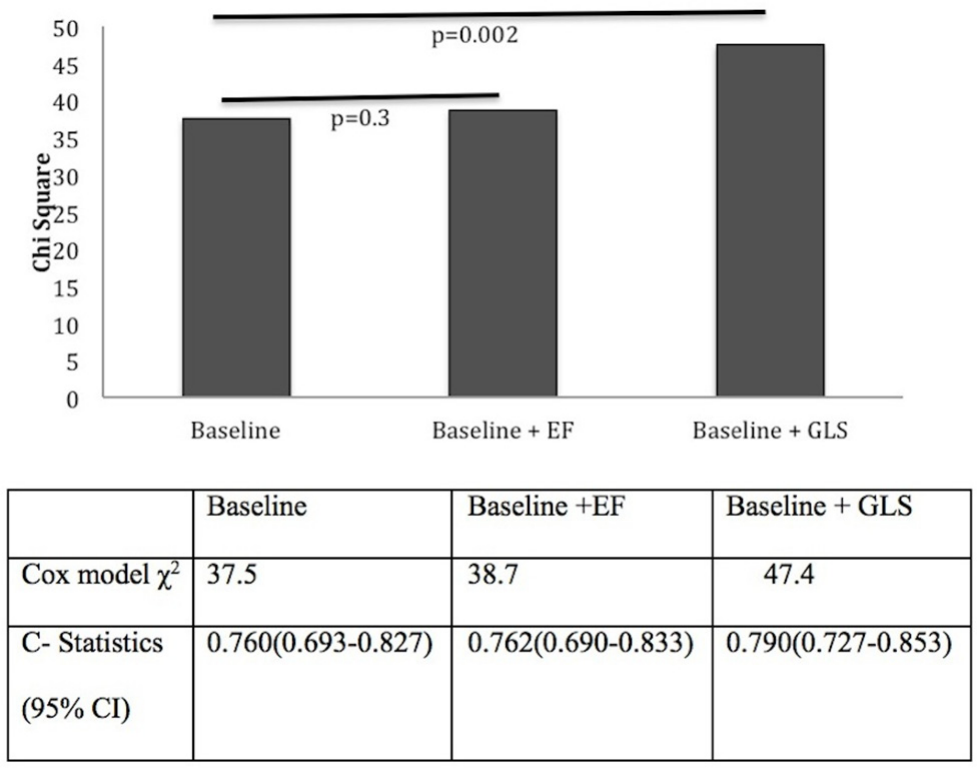
CV mortality: Comparing incremental value of EF and GLS to relevant demographic and biochemical variables (baseline model: age, gender, diabetes mellitus, smoking history, previous cardiovascular events, systolic BP).

The predictive power of GLS and EF was also assessed in separate nested Cox models to obtain additional prognostic information on each measure of LV function. Models adding EF or GLS to baseline models with relevant demographic and biochemical variables were compared (Figs 3 and 4). Addition of EF resulted in a very modest increase in predictive power [All-cause mortality model χ^2^ 70.7 to 71.6 (p = 0.3); CV mortality model χ^2^ 37.5 to 38.7 (p = 0.3)]. In contrast, addition of GLS resulted in a significantly greater increase in predictive power for both all-cause (model χ^2^ 70.7 to 80.5 p = 0.002) (Fig 3) and CV mortality (model χ^2^ 37.5 to 47.4, p = 0.002)(Fig 4). Similarly, discriminatory analysis with c-statistics showed the addition of GLS to baseline models improved risk prediction of all-cause mortality [0.727(95% CI 0.674–0.780) to 0.736 (95% CI 0.684–0.789)] and CV mortality [0.760 (95% CI 0.693–0.827) to 0.790 (95% CI 0.727–0.853)].

### Prognostic value of GLS in patients with preserved EF

**Fig 5:**
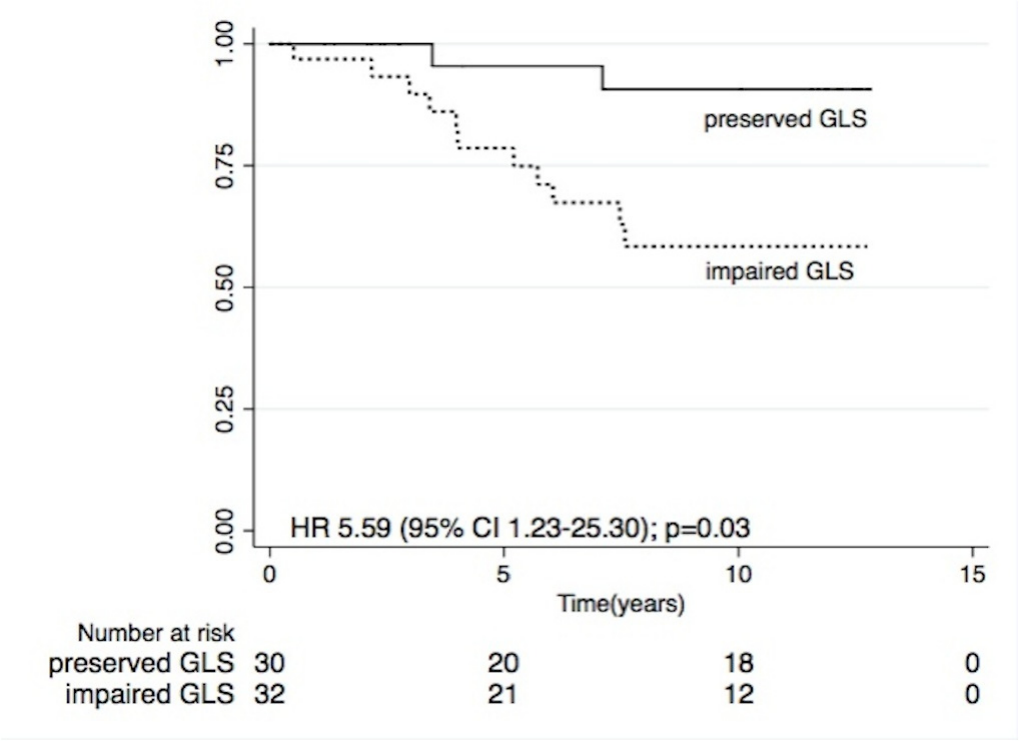
Kaplan Meier CV survival estimates according to GLS in patients with preserved EF.

A total of 62 patients (34%) had EF ≥ 50% (mean age 56± 15 years and 61% men). 51% of patients in this subgroup had impaired GLS (mean GLS -15.8± 3.8%). In univariate analysis, GLS was associated with increased cardiac death (HR 1.24 95% CI 1.04–1.49, p = 0.02) and impaired GLS was associated with a 5.6 fold increase in CV mortality (HR 5.59 95% CI 1.23–25.30, p = 0.03) in participants with preserved EF (Fig 5). However, there was no association between GLS and overall mortality in this subgroup. Due to the limited number of patients and events, we did not proceed to evaluate the independent association of GLS and CV mortality with multiple regression models.

### Sensitivity analyses

Sensitivity analysis using a competing risk regression model was used to delineate the confounding effect of renal transplantation on the outcomes. The association between GLS and all-cause and CV mortality remained after accounting for renal transplantation as a competing risk [all-cause mortality; GLS HR: 1.10 (95% CI 1.05–1.15) p<0.0001, CV mortality; HR: 1.14(95% CI 1.05–1.24) p<0.01]. Mortality rates, incident rate ratios and survival estimates were also repeated with an EF cut-off value of 45%. There was no difference in overall and CV survival outcomes if patients had EF<45% versus EF≥ 45%.

## Discussion

The present study demonstrated that worsening GLS was independently associated with a higher all-cause and CV mortality in patients with Stage 4, 5 and 5D CKD. The predictive value of GLS for survival was superior to other established CV risk factors and without confounding effects of receiving renal transplant. Our study also identified an association between impaired GLS (>-16%) and adverse CV outcome in patients with preserved left ventricular EF.

Few studies have assessed the prognostic value of myocardial strain assessment in CKD. We previously reported the relationship between GLS with renal impairment and all-cause mortality and found that GLS was a sensitive discriminator over significant clinical risk factors in predicting all-cause mortality[19]. In a cohort of 88 stable hemodialysis patients with preserved EF and a mean follow-up of 25.6 ± 9.9 months, Liu et al found an independent association between impaired GLS and all-cause mortality[35]. More recently, Kramann et al demonstrated the ability of strain parameters to identify uremic cardiomyopathy and predict CV mortality in dialysis patients[20]. The current study had a more inclusive cohort encompassing stable hemodialysis, peritoneal dialysis and pre-dialysis stage 4 and 5 CKD patients and involved a much longer duration of follow-up. This study not only confirmed the independent relationship between GLS with CV and all-cause mortality in patients with CKD but also highlights the effect of GLS on survival is independent and additive to established prognostic parameters such as EF and LVMI. Several observational and prospective studies in unselected populations with heart failure, myocardial infarction and cardiomyopathy[13–15, 17, 36] have identified prognostic value and superior risk stratification of GLS compared to EF [37]. In a recent study by Ersboll et al who assessed 548 patients with acute myocardial infarction (MI) within 48 hours of admission, GLS outperformed EF and biochemical measurement of N-terminal pro-brain natriuretic peptide, in predicting in-hospital heart failure[36]. In addition, these investigators have emphasized the independent prognostic value of GLS in predicting CV death and HF in patients with preserved EF[15, 36]. In a high-risk population with advanced CKD, our study found that impaired GLS was associated with an unadjusted 5.6-fold increased risk for CV mortality in patients with preserved EF. This intriguing finding warrants further evaluation as our observation was limited by a small number of participants in this subgroup. Furthermore, as HF is a common cause of CV death in patients with HF with preserved EF[38, 39], the ability to risk stratify these patients and compare GLS with other parameters of LV function requires further investigation with larger studies.

Another interesting finding of the current study is that 51% of patients with normal EF had impaired strain. Emerging evidence from general population studies has identified similar systolic abnormalities in patients with preserved EF. Ternacle et al found 40% of patients with preserved EF (defined as ≥50%) had an abnormal GLS (defined as >-16%)[16] in patients undergoing pre-operative assessment for cardiac surgery. Similarly, patients with heart failure and preserved ejection fraction (HFpEF) were shown to have significantly lower GLS compared to a control population[40]. Although GLS and EF are highly correlated, they measure different aspects of the myocardial deformation. EF predominantly quantifies radial contraction and GLS represents the function of subendocardial longitudinal myocardial fibres that are more sensitive to reduced coronary perfusion and increased wall stress.[41, 42]. GLS not only provides quantitative assessment of myocardial function but also reflects changes in the myocardial interstitium including extent of myocardial fibrosis[20, 43]. CKD is a unique risk factor for cardiac remodelling; animal models have demonstrated that early subendocardial changes are significantly worse in CKD compared to non-CKD rats following similar cardiac insults[44]. CKD specific disturbances such as hyperuricemia, abnormal bone mineral metabolism, pressure and volume overload have been shown to drive these mechanistic changes[45, 46].

Two studies have observed no difference in GLS measurements in relation to timing of hemodialysis [35, 47]. However, similar to EF, GLS has been shown to be sensitive to loading conditions especially afterload[48]. It is important to note that systolic BP is a major determinant of GLS[32] and should be meticulously included at the time of measurement. Thus, in this study, we adjusted for potential confounding effects of loading on strain by including LVEDV, LV wall stress and systolic BP. Variation in GLS measurements due to loading conditions among dialysis patients should be further examined. Nevertheless, GLS may yield greater detection of early LV dysfunction in patients with CKD.

This study has a number of strengths. It represents the largest study involving stable patients with advanced CKD (CKD 4/5, hemodialysis and peritoneal dialysis) to evaluate the prognostic significance of GLS and the study had a long follow-up of 7.8 ± 4.4 years. However the results of this study warrant careful interpretation due to some limitations: 1) we were unable to conduct a subgroup analysis according to stages of CKD or dialysis modality due to the relative small sample sizes in each group; 2) previous studies have shown relationship between EF and clinical outcomes[1], however in this study there was no differences in mortality between subjects with preserved and impaired EF. This discrepancy could be due to our sample size; 3) previous studies have also shown that diastolic dysfunction occurs in early stages of CKD and can predict adverse outcome[49, 50]; however in this study we did not have measures of diastolic function in majority of patients to directly compare prognostic relevance of systolic and diastolic function.; 4) we only conducted univariate analysis in patients with preserved EF due to the small number of events in this subset. Additional studies are required to identify the prognostic utility of GLS in CKD patients with diastolic dysfunction and in patients with preserved EF; 5) even though we adjusted for a large number of patient characteristics, the possibility of residual confounding also cannot be excluded; 6) we used resting echocardiogram and were unable to identify inducible ischemia as one of the predictors of mortality; 7) inter-vendor variability can affect GLS measurements, thus the prognostic value of GLS shown here may be limited to the equipment used in this study.

In conclusion, the present study demonstrated that GLS is associated with all-cause and cardiovascular mortality among patients with advanced CKD. Studies of larger populations in CKD are required to confirm that GLS provides additive prognostic value in patients with preserved EF.
